# Supplementary material for: Identification of photoperiod-induced specific miRNAs in the adrenal glands of Sunite sheep (Ovis aries)
Source: Front Vet Sci. 2022 Jul 22;9:888207. doi: 10.3389/fvets.2022.888207 (PMC9354845; doi:10.3389/fvets.2022.888207)
Supplement: Supplementary file 1 [file Data_Sheet_1.ZIP › Supplementary file/miRDeep2 file.zip!/miRDeep2 file/Novel_Structure/LP42A2/Novel_37.pdf]

Provisional ID	:	NC_019460.2_8176
Score total	:	2.4
Score for star read(s)	:	-1.3
Score for read counts	:	0
Score for mfe	:	2.1
Score for randfold	:	1.6
Score for cons. seed	:	
Total read count	:	161
Mature read count	:	159
Loop read count	:	0
Star read count	:	2

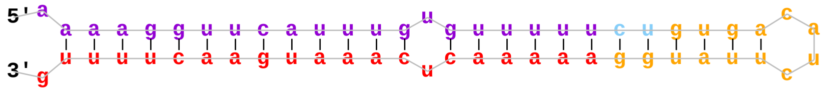

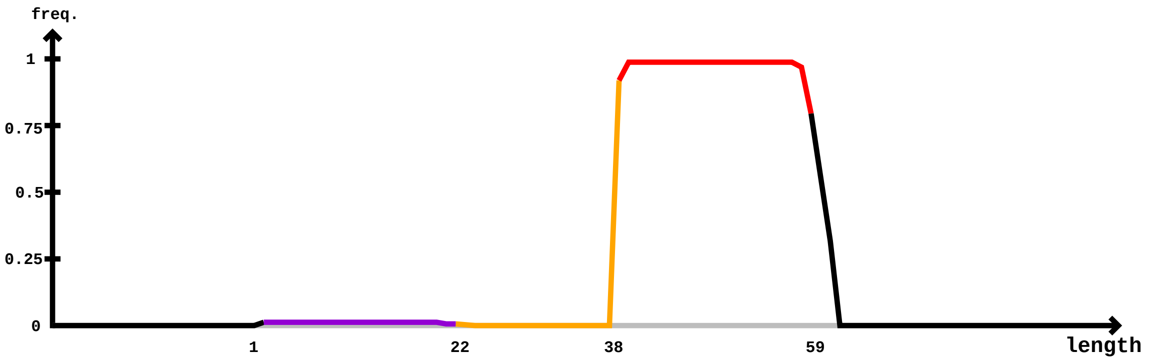

```
                    Star                                 Mature
5'- gaaauaaauacuggguuggccaaaagguucauuuguguuuuucugugacaucuuaugg aaaaacucaaaugaacuuuugggccaacccaaugaaaagcuccaaucccuuc   -3'   obs
    gaaauaaauacuggguuggccaaaagguucauuuguguuuuucugugacaucuuaugg aaaaacucaaaugaacuuuugggccaacccaaugaaaagcuccaaucccuuc         exp
    ...........((((((((((.(((((((((((((.((((((((((((....)))))))))))).))))))))))))).)))))))))).....................   reads   mm   sample
    .....................aaaagguucauuuguguuu......................................................................   1       0    seq
    .....................aaaagguucauuuguguuuuu....................................................................   1       0    seq
    ..........................................................aaaaacucaaaugaacuuu.................................   2       0    seq
    ..........................................................aaaaacucaaaugaacuuC.................................   1       1    seq
    ..........................................................aaaaacucaaaugaacuuuC................................   1       1    seq
    ..........................................................aaaaacucaaaugaacuuuA................................   1       1    seq
    ..........................................................aaaaacucaaaugaacuuuu................................   26      0    seq
    ..........................................................aaaaacucaaaugaacuuuuA...............................   51      1    seq
    ..........................................................aaaaacCcaaaugaacuuuug...............................   1       1    seq
    ..........................................................aaaaacucaGaugaacuuuug...............................   1       1    seq
    ..........................................................aaaaGcucaaaugaacuuuug...............................   3       1    seq
    ..........................................................Gaaaacucaaaugaacuuuug...............................   1       1    seq
    ..........................................................aaaaacucaaaugaacuuuuU...............................   1       1    seq
    ..........................................................aaaaacucaaaugaacuuuuUg..............................   9       1    seq
    ..........................................................aaaaacucaaaugaacuuuuggU.............................   42      1    seq
    ..........................................................aaaaacucaaaugaacuuuuggC.............................   8       1    seq
    ...........................................................aaaacucaaaugaacuuuug...............................   1       0    seq
    ...........................................................aaaacucaGaugaacuuuugg..............................   2       1    seq
    ...........................................................aaaacucaaaugaacuuuugg..............................   1       0    seq
    ...........................................................aaaacucaaaugaacuuuuUg..............................   6       1    seq
    ...........................................................aaaacucaaaugaacuuuuggU.............................   1       1    seq
```
